# Risk factors associated with stillbirth of piglets born from oxytocin-assisted parturitions

**DOI:** 10.14202/vetworld.2020.2172-2177

**Published:** 2020-10-17

**Authors:** Nguyen Hoai Nam, Peerapol Sukon

**Affiliations:** 1 Department of Animal Surgery and Theriogenology , Faculty of Veterinary Medicine, Vietnam National University of Agriculture, Trauqui, Gialam, Hanoi, Vietnam; 2 Department of Anatomy, Faculty of Veterinary Medicine, Khon Kaen University, Mueang Khon Kaen District, Khon Kaen 40002, Thailand; 3 Research Group for Animal Health Technology, Khon Kaen University, Mueang Khon Kaen District, Khon Kaen 40002, Thailand

**Keywords:** birth order, oxytocin, pig, ponderal index, stillbirth

## Abstract

**Aim:**

The present study aimed to investigate the effects of different risk factors on stillbirth of piglets born from oxytocin-assisted parturitions.

**Materials and Methods:**

Data were collected from a total of 1121 piglets born from 74 Landrace x Yorkshire crossbred sows from a herd. Logistic regression models were used to determine the associations between stillbirth and different risk factors including parity (1, 2, 3-5, and 6-10), gestation length (GL) (112-113, 114-116, and 117-119 days), litter size, birth order (BO), sex, birth interval (BI), cumulative farrowing duration, birth weight (BW), crown rump length, BW deviation, body mass index, ponderal index (PI), and the use of oxytocin during expulsive stage of farrowing.

**Results:**

The incidence of stillbirth at litter level and stillbirth rate was 59.5% (44/74) and 8.1% (89/1094), respectively. The final multivariate logistic regression selected BO, BI, PI, GL, and parity as the five most significant risk factors for stillbirth. Increased BO and BI, GL <114 and >116 days, parity 6-10, and low PI increased the stillbirth rate in piglets.

**Conclusion:**

Several factors previously determined as risks for stillbirth in exogenous oxytocin-free parturitions also existed in exogenous oxytocin-assisted parturitions. One dose of oxytocin at fairly high BO did not increase stillbirth, whereas two doses of oxytocin were potentially associated with increased values.

## Introduction

Oxytocin is commonly used as an obstetric intervention to reduce farrowing duration in sows [1], with controversial effects on stillbirth rates. Some authors did not find any correlation between the use of oxytocin and stillbirth incidents [2-5], while other researchers reported that oxytocin reduced stillbirth rates [6]. On the other hand, there are many studies that found increased stillbirths due to oxytocin-induced asphyxia [7-11].

Studies that evaluated the effect of oxytocin on stillbirth at litter level [4,5,12] might include some degree of bias since stillborn piglet(s) might be born before the use of oxytocin. At the same time, evaluation of effect of oxytocin on stillbirth is difficult when it is combined with manual extraction [4,12,13]. Administration of oxytocin after the birth of the first piglet resulted in either increased hypoxia and stillbirth [7-11] or non-significant effect [14]. Although those studies significantly contributed in the understanding of oxytocin action in uterus and of its effect on stillbirth, the administration of oxytocin at the very early stages of parturition did not resemble the real practice at farrowing condition. There is also a study that examines the relationship between oxytocin use at different birth order (BO) (after 1, 4, and 8) and stillbirth incidents [15], but the interaction of oxytocin use with other factors was not evaluated despite the fact that stillbirth has a multifactorial cause. To the best of our knowledge, a study assessing the effect of sow, farrowing, and piglet traits on stillbirth of piglets born from exogenous oxytocin-assisted farrowed sows has not been reported.

The aim of this study was to investigate the effects of different factors including parity, gestation length (GL), litter size (LS), piglet gender, birth interval (BI), cumulative farrowing duration (CFD), BO, crown rump length (CRL), birth weight (BW), birth weight deviation (BWD), body mass index (BMI), and ponderal index (PI) on the stillbirth of piglets born from exogenous oxytocin-assisted parturitions.

## Materials and Methods

### Ethical approval

This study did not use animal samples or involve activities that could harm the investigated animals.

### Study period, location, animals and housing

This study was conducted from February to May 2019; included a total of 1121 piglets born from 74 sows on a swine farm in Phu Tho province in the North of Vietnam. The farm had a breeding herd of 600 sows. During gestation, sows were housed in individual crates sized 60 cm width × 220 cm length. Approximately a week before the estimated farrowing date, animals were removed to individual farrowing crates sized 180 cm width × 220 cm length. At farrowing crates animals were allocated into a slatted floor area which measured 60 cm × 220 cm. The farrowing crates were cleaned twice per day. Sows were bathed once per day or every 2 days depending on the ambient temperature. The temperature at the farrowing room ranged between 20°C and 26°C.

Sows were fed 3 times/day. During the first 84 days of gestation, sows were provided with 1.8-2.5 kg of feed per day containing 13% of crude protein and metabolizable energy of 2900 kcal/kg (Hi-Gro 566F, Charoen Pokphand, Vietnam). In the last trimester, sows were daily received 3-3.5 kg of feed containing 17% crude protein and metabolizable energy of 3100 kcal/kg (Hi-Gro 567SF, Charoen Pokphan, Vietnam). At parturition, sows were deprived of feed. After parturition, amount of feed was increased gradually from 1.5 kg on day 1 to about 6.5 kg on day 6. Water was provided *ad libitum* through a bite nipple system.

### Data collection

Sows were supervised continuously at least from the birth of the first piglet to the birth of the last piglet. All the born piglets were handled by one veterinarian. Parity number was recorded from sow’s card. GL was the interval between the date of the first insemination and the farrowing date. At the birth of each piglet the sex of piglet, birth time and BO were recorded. The BI (min) was defined as the interval between the births of two successive piglets, and therefore the first piglets did not have a BI. The CFD (min) was calculated as the interval between the birth of a given piglet and the birth of the first piglet, the CFD of the first piglets of all litters was 0. Stillbirth examination was conducted immediately after the piglet expulsion. Stillborn piglets were defined as piglets died before the expulsion without any sign of decay [3]. Stillbirth rate was the proportion of all stillborn piglets to the sum of born alive and stillborn from all litters. Birth LS was calculated as the total number of born alive, stillborn, and mummified piglets. Mucus was suckled from piglets’ mouth and nose, and their bodies were dried by hygroscopic flour (Safeguard, Greenvet, Vietnam). Piglets’ BW (kg) was measured individually using a 5 kg scale (Nhon Hoa, Vietnam). CRL (cm) of piglets was measured using a tape measure (Vietnam). The measurement of BW and CRL was handled gently to minimize stress, and lasted about 35-40s for each piglet. BWD (kg) was defined as the difference between BW of a given piglet and the mean of individual BW of piglets (alive and stillborn) in that litter. Piglets’ BMI was calculated using the following equation: BW (kg)/(CRL, m)^2^ [16]. Piglets’ PI was calculated using the following equation: BW (kg)/(CRL, m)^3^ [16]. During the expulsive stage, all the sows were injected 1-2 doses of oxytocin (20UI, CP-CIN 20, L.B.S. Laboratory L.T.D., Thailand) by farm’s workers. The veterinarian recorded the BO at which oxytocin was used.

### Statistical analysis

Descriptive statistics were generated using all available data of sows and piglets. Risk analysis was conducted in piglets with full records resulting in removal of the 74 first-born piglets that did not have a BI, 27 mummified piglets and 25 piglets with missed information in other criteria. Consequently, 995 piglets with the full record were included in risk analyses. Potential risk factors for stillbirth included oxytocin use (piglets born before oxytocin administration [O_0], born after one dose of oxytocin [O_1], and born after 2 doses of oxytocin [O_2]), parity (1, 2, 3-5, and 6-10), GL (112-113, 114-116, and 117-119 days), LS, BO, piglet gender, CRL, BW, BWD, BI, CFD, BMI, and PI. The binary dependent variable was stillbirth status of individual piglet which was categorized into No stillbirth or Stillbirth.

The relationship between 13 potential risk factors for stillbirth was analyzed using Pearson’s bivariate correlation (IBM SPSS Statistics for Windows, Version 22.0, IBM Corp., Armonk, NY, USA).

A two-step modeling approach was used to determine potential risk factors associated with stillbirth. At first, a univariate logistic analysis was performed to quantify the correlation between stillbirth status and each risk factor. Second, the variables with p ≤ 0.1 in the univariate analysis were further analyzed in the multivariate analysis. A forward stepwise model building method using Akaike information criterion as the calibration tool was employed to select the final model. All statistical analyses were carried out in STATA, version 15 (StataCorp LP, College Station, TX: StataCorp LLC). A p ≤ 0.05 was set as the statistical significance level in the final model.

## Results

Forty-four (59.5%) out of 74 litters had at least one stillborn piglet. Among them, 22 litters had 1 stillbirth, 12 litters had 2 stillbirths, and 10 litters had more than 2 stillbirths. Among the 1121 piglets born to 74 litters, 27 piglets were mummified (2.4%) and 89 piglets were stillborn (8.1%, 89/1094). Sixty-four (86.5%) sows were injected with 1 dose of oxytocin, and 10 (13.5%) sows were injected with two doses of oxytocin during the stage of fetal expulsion. The average and median BO at which the first and the second dose of oxytocin was injected was 8.2±3.7 and 8, and 10.9±2.9 and 11, respectively. Other parameters of investigated sows and piglets are shown in Table-1.

**Table 1:** Descriptive parameters of investigated sows and piglets.

Parameters	Number	Mean±SD
Parity	74	4.6±2.2
GL	74	115.4±1.2
LS	74	15.8±3.1
CRL	1081	27.0±2.6
BW	1072	1.27±0.44
BI	1013	19.0±41.7
CFD	1087	149.5±141.9
BMI	1072	17.1±4.7
PI	1072	63.3±17.2
BWD	1072	0.0003±0.36886

SD=Standard deviation, GL=Gestation length (day), LS=Litter size, CRL=Crown rump length (cm), BW=Birth weight (kg), BI=Birth interval (min), CFD=Cumulative farrowing duration (min), BMI=Body mass index, PI=Ponderal index, BWD=Birth weight deviation (kg)

The significant correlations between significant risk factors for stillbirth are demonstrated in Table-2.

**Table 2:** Matrix of significant correlations between the significant risk factors regarding stillbirth.

	LS	BW	BWD	PI	BMI	BO	BI	CFD	O
BW	−0.205**								
BWD		0.834**							
PI	−0.112**	0.670**	0.537**						
BMI	−0.162**	0.875**	0.718**	0.944**					
BO	0.328**		0.080*	−0.071*					
BI	−0.064*					−0.109**			
CFD	0.172**	−0.064*		−0.174**	−0.141**	0.469**	0.256**		
O	0.195**			−0.127**	−0.092**	0.650**		0.582**	
P	0.075*							−0.064*	−0.075*
GL								−0.150**	

LS=Litter size, BW=Birth weight, BWD=Birth weight deviation, PI=Ponderal index, BMI=Body mass index, BO=Birth order, BI=Birth interval, CFD=Cumulative farrowing duration, O=Use of oxytocin, P=Parity, GL=Gestation length.

* means the correlation is significant at the 0.05 level.

** means the correlation is significant at the 0.01 level

Univariate analysis results of potential risk factors for stillbirth in piglets are presented in Table-3. All risk factors, apart from sex and CRL, were significantly associated with stillbirth.

**Table 3:** Results of univariate analysis of risk factors for stillbirth (995 piglets).

Covariates	Stillbirth rate	OR; 95 CI	Nagelkerke R2	p-value
Born before the use of O	3.9 (20/511)	1	0.0791	
Born after 1 dose of O	10.5 (45/433)	2.85; 1.65-4.90	<0.001	
Born after 2 doses of O	31.4 (16/51)	11.22; 5.35-23.56	<0.001	
Parity 6-10	11.7 (42/360)	1	0.0285	
Parity 2	1.2 (1/86)	0.09; 0.01-0.66	0.018	
Parity 1	5.0 (6/121)	0.40; 0.16-0.95	0.039	
Parity 3-5	7.5 (32/428)	0.61; 0.10-0.18		0.046
GL=114-116 days	6.9 (52/751)	1	0.0155	
GL=117-119 days	9.4(16/170)	1.40; 0.78-2.51		0.265
GL=112-113 days	17.6 (13/74)	2.86; 1.48-5.55		0.002
Female	8.0 (36/450)	1	<0.001	
Male	8.3 (45/545)		1.035; 0.66-1.63	0.883
LS		1.08; 1.007-1.16	0.082	0.032
BO		1.19; 1.13-1.25	0.0834	<0.001
BI		1.007; 1.003-1.01	0.0239	<0.001
CFD		1.004; 1.003-1.006	0.0594	<0.001
BW		0.92; 0.88-0.97	0.0167	0.002
CRL		1.02; 0.93-1.11	0.0003	0.681
BWD		0.39; 0.21-0.70	0.0174	0.002
BMI		0.90; 0.85-0.94	0.0352	<0.001
PI		0.97; 0.95-0.98	0.042	<0.001

OR=Odds ratio, CI=Confident interval, p=Probability level, O=Oxytocin, GL=Gestation length, LS=Litter size, BO=Birth order, BI=Birth interval, CFD=Cumulative farrowing duration, BW=Birth weight, CRL=Crown rump length, BWD=Birth weight deviation, BMI=Body mass index, PI=Ponderal index

The final model, with an overall significant value of 0.001, showed that BO, PI, BI, GL, and parity were the five most important risk factors with respect to stillbirth in the piglets (Table-4). The final model explained 21.53% variation of stillbirth in piglets. BO was the most significant factor explaining 8.34% variation of stillbirth. PI, BI, GL, and LS explained 3.42, 3.00, 1.84, and 4.93% variation of stillbirth, respectively. The Hosmer-Lemeshow goodness of fit test showed good fit between expected and predicted outcome (p=0.778).

**Table 4:** Results of multivariate analysis of risk factors for stillbirth (995 piglets).

Stillbirth	OR; 95%CI	Nagelkerke R2 change	p-value
BO	1.21; 1.14-1.28	0.0834	<0.001
PI	0.96; 0.94-0.98	0.1176	<0.001
BI	1.01; 1.00-1.01	0.1476	<0.001
GL=114-116 days	1	0.1660	
GL=117-119 days	2.12; 1.10-4.08		0.024
GL=112-113 days	5.50; 2.50-12.07		<0.001
Parity=6-10	1	0.2153	
Parity=2	0.03; 0.004-0.26		0.001
Parity=1	0.32; 0.12-0.84		0.021
Parity=3-5	0.46; 0.27-0.78		0.004

OR=Odds ratio, CI=Confident interval, p=Probability level, BO=Birth order, PI=Ponderal index, BI=Birth interval, GL=Gestation length. The final model selected BO, PI, BI, GL, and parity as the five most significant factors for stillbirth in piglets. The overall significance of final model was 0.001. The final model explained 21.53% variation of the stillbirth. The Hosmer-Lemeshow goodness of fit test showed a good fit between expected and predicted outcome (p=0.778)

The final model rejected CFD and O in favor of BO to be a better indicator regarding stillbirth. In all models containing both BO and O, the stillbirth rate in piglets born before the use of oxytocin was not different from that in piglets born after the use of 1 dose of oxytocin, but different from that in piglets born after the use of 2 doses of oxytocin. Piglets with lower BW and BMI were more likely to be stillborn than those with higher BW and BMI, respectively. However, PI was more significant than BW and BMI with respect to explanation of stillbirth variation and was retained in the final model. PI was negatively associated with stillbirth (p<0.001), whereas increased BI resulted in increased stillbirth (p=0.001). Piglets born from sows with a GL of 114-116 days were less likely to be stillborn in comparison with piglets born from sows with a GL of 112-113 days (p<0.001) and 117-119 days (p=0.024). Piglets born from sows at parity 1, 2, and 3-5 had lower possibility of being stillborn compared to piglets born from sows at parity 6-10 (OR=0.32 [p=0.021], OR=0.03 [p=0.001], and OR=0.46 [p=0.004]), respectively.

## Discussion

The stillbirth rate (8.1%) found in this study is higher than that previously reported by many other authors [14,17-20]. The proportion of litters having stillbirth(s) (59.5%) was also higher than that in any previously published articles [3,5,17,21].

The direct relationship between stillbirth and BO found in the present study is in accordance with the existing literature [16,22]. This study also confirms the finding of Baxter *et al*. [23] that BO is more important than CFD in prediction of stillbirth. It was reported that the stillbirth rate was increased from 2% in the first-born piglets to 17% in the piglets born 13^th^ or later [24]. The result was quite similar to the findings of the present study where stillbirth rate was 3.4% till the birth of the 5^th^ piglet, and increased up to 12.8% between the 11^th^ and 15^th^ piglet and to 21.4% after the 15^th^ piglet. Piglets with a long CFD did not necessarily have a high BO, therefore, did not necessarily experience a high number of uterine contraction series. In contrast, piglets with a high BO were more likely to be subjected to an increased number of uterine contraction series, thereby being predisposed to higher risks of distress and hypoxia and subsequently being stillbirth.

The present study also confirms previously published results [16,22] that BI is an important indicator of stillbirth in piglets. The finding that BI is more important than CFD regarding stillbirth is in agreement with that of Baxter *et al*. [22]. On the other hand, Langendijk *et al*. [25] suggested that interventions aiming at reducing stillbirth rate should focus on CFD rather than BI because the stillbirth risk significantly increased only when a BI exceeded 90 min, whereas the stillbirth possibility started to increase when a CFD exceeded 120 min. However, in the present study, the risk of stillbirth significantly elevated at a BI >60 min (7.5%, 7.1%, 8.3%, and 20.4% at BI <20, 21-40, 41-60, and >60 min, respectively), and at a CFD >300 min (4.7%, 8.1%, 7.7%, and 21.5% at CFD <120, 121-240, 241-300, and >300 min, respectively). At present, there are no clear explanations for the increased importance of BI over CFD regarding stillbirth prediction. BI more suitably represents the time period that a given piglet actively manages to go through the cervix and vagina to the outside environment, whereas CFD represents the cumulative time period a given piglet is less active in the uterus plus a BI. There is no information about the different pressure levels that a piglet has to experience at different locations of the birth canal. However, based on the superior tension and thickness of the cervix and its smaller size in comparison with uterus and vagina, and its inherent long size, it can be hypothesized that a fetus experienced the highest pressure when it is pushed through the cervix. Alonso-Spilsbury *et al*. [26] reported that injection of oxytocin after the birth of the first piglet resulted in 70.8%, 8.3%, and 20.8% stillbirths at BO <5, 5-8, and ≥9, respectively. This result implied the increased important role of BI compared with that of CFD in explaining stillbirth, and that may be a possible explanation to the selection of BI rather than CFD in the final model with respect to stillbirth prediction.

Body size and shape have been widely recognized as some of the most important indicators for piglet stillbirth [16,22,23]. The previous studies suggested that PI and BMI were more important indices than BW and CRL [16,23]. The present study also confirmed that PI, among conformation traits, was the most significant factor with respect to stillbirth. Piglets with a low PI tend to have a low BW with either a normal or a longer-than-normal CRL. The disproportionate piglets were suggested to be suffered from uterine growth retardation [27]; therefore, they did not have good health to achieve a good chance of prenatal survival. Furthermore, the proportionately small piglets have lower hemoglobin level in comparison with heavier littermates [28]. During farrowing process, distress, and hypoxia are not uncommon [8]; therefore, the lower hemoglobin level becomes a disadvantage for the small piglets and as the consequence, they were more likely to be stillborn.

There are controversies over the effect of GL on stillbirth of piglets. Some authors did not detect any effect of GL on stillbirth [29]. In contrast, the negative association between short GL and stillbirth has been reported [17]. Piglets born from a short GL associated with immaturity [28] and, therefore, acquired higher risk of being stillbirth. On the other hand, increased stillbirth in human has been also found in prolonged gestation [30-32], and this has been suggested to be attributable to placental insufficiency [33]. Therefore, the increased possibility of delivering stillborn piglets by sows with GL=117-119 days may be an effect of placental insufficiency attributable to prolonged gestation.

The observed effect of parity number on stillbirth, in this study, was highly similar to that of Vanderhaeghe *et al*. [34]. In that study, the stillbirth rate in piglets born from sows at parity 1, 2, 3-6, and >6 was 6.8, 6.1, 7.4, and 10.3%, respectively. It was suggested that prolonged farrowing was the reason of increased stillbirth rates in high parity [5]. This fact was also demonstrated in the present study because BI and CFD of piglets born from sows at their 6^th^-10^th^ parity were significantly longer than that of piglets born from 1, 2, and 3-5 parity sows (22.1 versus 16.4, 15.6, and 17.7 min [BI], and 176.6 versus 113.5, 126.6, and 158.0 min [CFD]).

The fact that the effect of the single dose of oxytocin on stillbirth rate found in univariate analysis disappeared in all the multivariate models containing BO and O suggested that the association between the use of one dose of oxytocin and stillbirth was the result of increased BO instead of oxytocin use itself. The harmful effect of exogenous oxytocin on stillbirth has been reported in previous studies [7,9-12], where oxytocin was administered immediately after the birth of the first piglets. At early parturition, the use of oxytocin may produce uterine hyperstimulation resulting in severe fetal distress and asphyxia [15]. In contrast, oxytocin administration after the birth of the 8^th^ piglet resulted in the improvement of the piglet survival through sufficient stimulation of uterine contraction without considerably decreased uterine blood flow [15]. In this study, oxytocin-hyperstimulation might not be induced since oxytocin was used at fairly high BOs (8.2±3.7 with median=8 for the first dose), which may be a possible explanation to the non-significant difference in stillbirth rate between piglets born before and after the use of 1 dose of oxytocin. On the other hand, the rejection of O in favor of other factors in the final model did not exclude potential effect of the use of two doses of oxytocin on stillbirth since this effect was significant in all models even with the presence of both BO and O. This effect can be explained by the fact that piglets born late in the farrowing process were subjected to a longer period of distress and hypoxia, and at very late parturition even mild distress and hypoxia caused by the second oxytocin injection could lead to an increased risk of fetal death.

## Conclusion

This study confirmed that several established risk factors for stillbirth in exogenous oxytocin-free parturitions also existed in exogenous oxytocin-assisted-parturitions. Increased BO and BI, parity 6-10, GL of 112-113 and 117-119 days, and low PI were the most significant factors associated with increased stillbirth. Injection of one dose of oxytocin at fairly high BO did not cause any significant effect on stillbirth; however, using two doses of oxytocin during the expulsive stage potentially increased stillbirth.

## Authors’ Contributions

NHN collected the data. NHN and PS conceived and designed the study, analyzed data, interpreted results, and wrote the manuscript. Both authors read and approved the final manuscript.
